# CD20-positive peripheral T-cell lymphoma not otherwise specified: a case series and review of the literature

**DOI:** 10.3389/fonc.2025.1553969

**Published:** 2025-04-09

**Authors:** Pan Zhou, Lifu Wang, Xiaohang Pei, Liu Yang, Yunmeng Zhou, Rongjun Ma, Yuqing Chen, Zunmin Zhu, Xiaoli Yuan

**Affiliations:** ^1^ Department of Hematology, Henan Provincial People’s Hospital, Zhengzhou University People’s Hospital, Zhengzhou, China; ^2^ Department of Pathology, Henan Provincial People’s Hospital, Zhengzhou University People’s Hospital, Zhengzhou, China

**Keywords:** peripheral T-cell lymphomas not otherwise specified, CD20, whole exome sequencing, prognosis, rituximab

## Abstract

Peripheral T-cell lymphoma not otherwise specified (PTCL-NOS) is a heterogeneous and aggressive malignancy that usually lacks B-cell associated antigens. Here, we identified three cases of PTCL-NOS that uniformly expressed T-cell specific antigens (CD2 and CD3) and the B marker CD20 (CD20+ PTCL-NOS). Molecular studies showed clonal rearrangement of the chain T-cell receptor genes without evidence of a clonal rearrangement of the immunoglobulin gene. All three patients were diagnosed with the advanced stages and had bone marrow (BM) involvement, of which one patient was confirmed by PET-CT and the other two patients were confirmed by BM biopsy. However, we surprisingly found that BM-infiltrated lymphoma cells did not express CD20. Whole-exome sequencing analysis revealed that the most common mutations were on the *DDX3X* and *TET2* genes. Although all the patients had relatively low Ki67 indices, they achieved poor response to the first-line treatment with CHOP-like chemotherapy. Subsequently, one patient was treated at a local hospital with an unspecified regimen, and the other two patients ultimately received rituximab combined chemotherapy as a salvage treatment after receiving multiple second-line therapies. However, the outcomes of these patients remained unsatisfactory. Therefore, it currently appears to be quite challenging to provide appropriate and effective treatment for patients with CD20+ PTCL-NOS.

## 1 Introduction

Peripheral T-cell lymphoma (PTCL) is a relatively uncommon, heterogeneous, mature T-cell malignancy. PTCL–not otherwise specified (PTCL-NOS) is one of the most common forms lacking pathognomonic features and does not correspond to any of the distinct entities listed in the WHO Classification of Tumours of Haematopoietic and Lymphoid Tissues (1). Usually, the morphological characteristics of PTCL-NOS are extremely variable, ranging from sheets of intermediate- to large-sized cells that are relatively devoid of an inflammatory environment to those that are polymorphous and enriched for a range of inflammatory cells (2). PTCL-NOS generally shares positive expression of T-cell-specific antigens (UCHL-1, CD2, CD3, CD5, and CD7), with negative expression of B-cell antigens (CD19, CD20, CD79a, and PAX5). Furthermore, CD20 is a classical pan-B-cell marker that has been rarely reported in diverse T-cell lymphoma, of which PTCL-NOS is classified as the majority subtype. This aberrant CD20 expression in PTCL-NOS may lead to a misdiagnosis as B-cell non-Hodgkin lymphoma (3, 4). PTCL-NOS generally demonstrates an aggressive clinical course and has inferior outcomes with a poor response to standard therapy. Nevertheless, due to its rarity, little is known about the prognostic impact of CD20 in PTCL-NOS. Recently, PTCL-NOS exhibited recurrent genetic alterations involved in epigenetic modifiers and T-cell receptor signaling pathway intermediates (5). However, the biological and clinical significance and genetic difference of CD20+ PTCL-NOS are unclear. Herein, we summarized the clinical and laboratory findings of three patients with CD20+ PTCL-NOS and additionally reviewed the literature to provide a more comprehensive understanding of this disease.

## 2 Case presentation

### 2.1 Clinical features and survival

**Figure 1:**
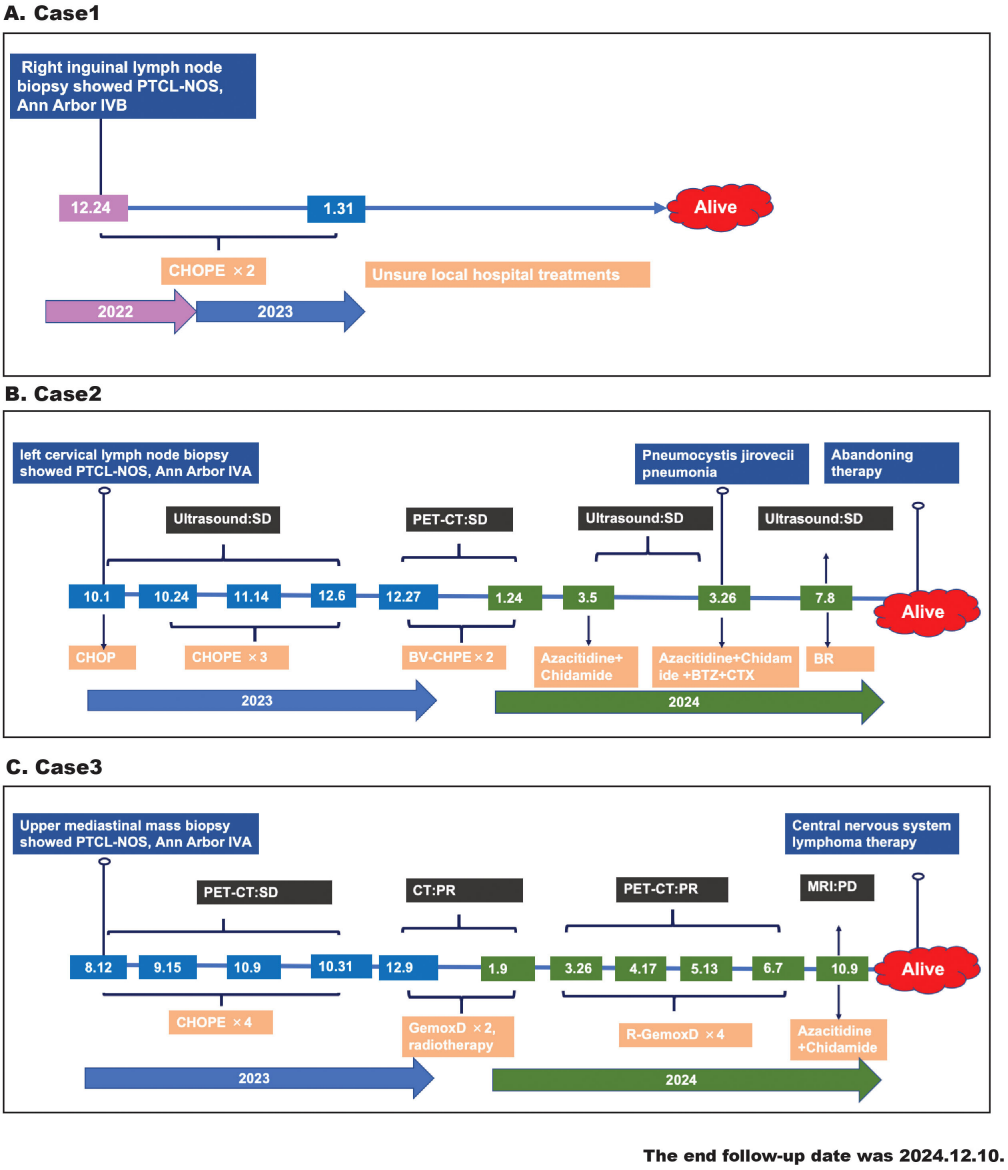
The timeline diagram of patients’ treatment history, response evaluation, and survival follow-up. **(A)** Case 1, **(B)** case 2, **(C)** case 3.

The clinical features of three patients with CD20+ PTCL-NOS are summarized in **Table 1** and **Figure 1**.

**Table 1:** Clinical features of CD20+ PTCL-NOS.

Case	Age	Sex	Clinical presentation	B symptoms	ECOG	Stage	IPI score	BM involvement	LDH	Treatments and response	Follow-up
1	69	Male	Abdominal distension, splenomegaly	Present	1	IV	2	Present (Bone marrow biopsy)	215U/L	CHOPE×2(PR), Unclear	Alive, PFS:23m, OS:23m
2	48	Male	Splenomegaly	Absent	0	IV	1	Present (Bone marrow biopsy)	184U/L	CHOP×1,CHOPE×3(SD),BV+CHPE×2(SD),Azacitidine+Chidamide,Azacitidine+Chidamide+BTZ+CTX(SD),BR(SD)	Alive, PFS:14m, OS:14m
3	61	Female	Chest distress, cough, mediastinal mass	Absent	1	IV	1	Present (PET/CT)	240U/L	CHOPE×4(SD),GemoxD×2, Radiotherapy (PR),R-Gemox-D×4(PR),Azacitidine+Chidamide(PD)	Alive, PFS:14m, OS: 16m

ECOG, Eastern Cooperative Oncology Group performance; IPI, International Prognostic Index; BM, bone marrow; LDH, lactic dehydrogenase; C, cyclophosphamide; H, doxorubicin; O, vincristine; P, prednisone E, etoposide; BV, brentuximab vedotin; BTZ, bortezomib; BR, bendamustine, rituximab; GemoxD, gemcitabine, oxaliplatin, dexamethasone; R, rituximab; PR, partial remission; SD, stable disease; PD, progressive disease; m, months.

Case 1 was a 69-year-old man who presented with a 6-month history of abdominal distension and weight loss. His general medical history revealed no special diagnoses. His Eastern Cooperative Oncology Group (ECOG) performance status was 1. The patient had palpable lymph nodes in the cervical, axillary, and inguinal regions and splenomegaly. The laboratory data showed mild leukopenia (3.24×10^9^/L), anemia (112g/L), decreased vitamin B12 (164pg/mL), and normal lactic dehydrogenase (LDH), and was negative for human immunodeficiency virus (HIV), hepatitis C virus (HCV), hepatitis B virus (HBV), cytomegalovirus (CMV) , epstein-barr virus (EBV) infections. He underwent a right inguinal lymph node biopsy, and the pathological diagnosis was confirmed as CD20+ PTCL-NOS. The bone marrow biopsy verified lymphoma infiltrating the bone marrow. Although the patient did not undergo PET-CT for financial reasons, he was evaluated to be in an advanced stage (Ann Arbor stage IVB) and achieved partial remission after two cycles of CHOPE (cyclophosphamide, doxorubicin, vincristine, prednisone, and etoposide). Afterwards, he continued his treatment at the local hospital with an unspecified regimen and survived to a follow-up time of almost 23 months.

Case 2 was a 48-year-old man with a left abdominal mass for 1 month without B symptoms. He did not have any history of illness, and his ECOG performance status was 0. A physical examination revealed cervical, axillary, and inguinal lymphadenopathy and splenomegaly. Laboratory findings showed elevated β2-microglobulin but normal complete blood cell count and LDH and were negative for HIV, HCV, HBV, CMV, and EBV infections. The PET-CT demonstrated increased metabolism in splenomegaly and multiple lymph nodes involving the bilateral neck, submandibular, clavicular, axillary, mediastinal, parapancreatic, hepatogastric hiatus, pedicle of the diaphragm, porta hepatis, retroperitoneum, mesentery, bilateral iliac fossa, and inguinal regions. He then underwent left cervical lymph node resection, and immunohistochemical (IHC) staining confirmed the diagnosis of CD20+PTCL-NOS. A bone marrow biopsy showed the involvement of lymphoma. The patient was classified as Ann Arbor stage IVA and received four cycles of chemotherapy (CHOP×1, CHOPE×3). Due to the poor response (stable disease) to the above regimens and positive CD30 expression, he was administered two cycles of brentuximab vedotin combined CHPE but continued to have stable disease, as evaluated by PET-CT. Yu J et al. reported that an epigenetic priming regimen with azacitidine (Aza) plus chidamide (Chi) showed promising anti-tumor effectiveness with a manageable safety profile in relapsed/refractory PTCL (RR-PTCL) patients (6). Moreover, bortezomib-based therapies were also considered as salvage regimens for RR-PTCL (7). Thus, he received one cycle of Chi+Aza and one cycle of Chi+Aza+BCD (bortezomib, cyclophosphamide, and dexamethasone). After this treatment, the patient still exhibited stable disease but developed a severe complication of *Pneumocystis carinii* pneumonia. After a long recovery, he restarted the anti-lymphoma treatment with the BR (bendamustine, rituximab) regimen on account of his positive CD20 expression. Due to the ineffectiveness of these treatments, the patient discontinued the anti-lymphoma treatment and waited for observation. The patient has survived for more than 14 months from the onset of the disease.

Case 3 was a 61‐year‐old woman with a chief complaint of chest distress and cough for 6 months. The patient did not have any B symptoms and denied any disease history. Physical examinations revealed a large subcutaneous mass in the left supraclavicular fossa. Her ECOG performance status was 1. Laboratory tests revealed elevated uric acid and low albumin but normal complete blood cell count and LDH, and were negative for HIV, HCV, HBV, CMV, and EBV infections. The PET-CT report showed lymphoma infiltrations involving the right side of the thoracic inlet trachea, the mediastinal region 2R-4R, mediastinal region 7, the right clavicular region’s multiple lymph nodes, and the left lower tibial medullary cavity. The tracheoscopic biopsy of her superior mediastinal mass confirmed the diagnosis of CD20+ PTCL-NOS. However, the iliac bone marrow biopsy was negative for malignant lymphoma cells. The patient was classified as Ann Arbor stage IVA and initially treated with four cycles of CHOPE. The following PET-CT showed a stable disease. GemoxD (gemcitabine, oxaliplatin, dexamethasone) has been reported as an effective salvage therapeutic option for RR-PTCL (8). Therefore, she was administered two cycles of GemoxD combined with irradiation and then achieved a partial remission, as assessed by CT. Based on the positive expression of CD20 and the unsatisfactory clinical responses, she received another four cycles of GemoxD combined with a rituximab regimen. However, the subsequent PET-CT indicated that the treatment response after R-GemoxD remained poor, and she was finally administered the Chi+Aza regimen. Although she had survived for more than 16 months after diagnosis, she experienced disease progression (central nervous system lymphoma) within 14 months.

### 2.2 Immunophenotypic features

**Figure 2:**
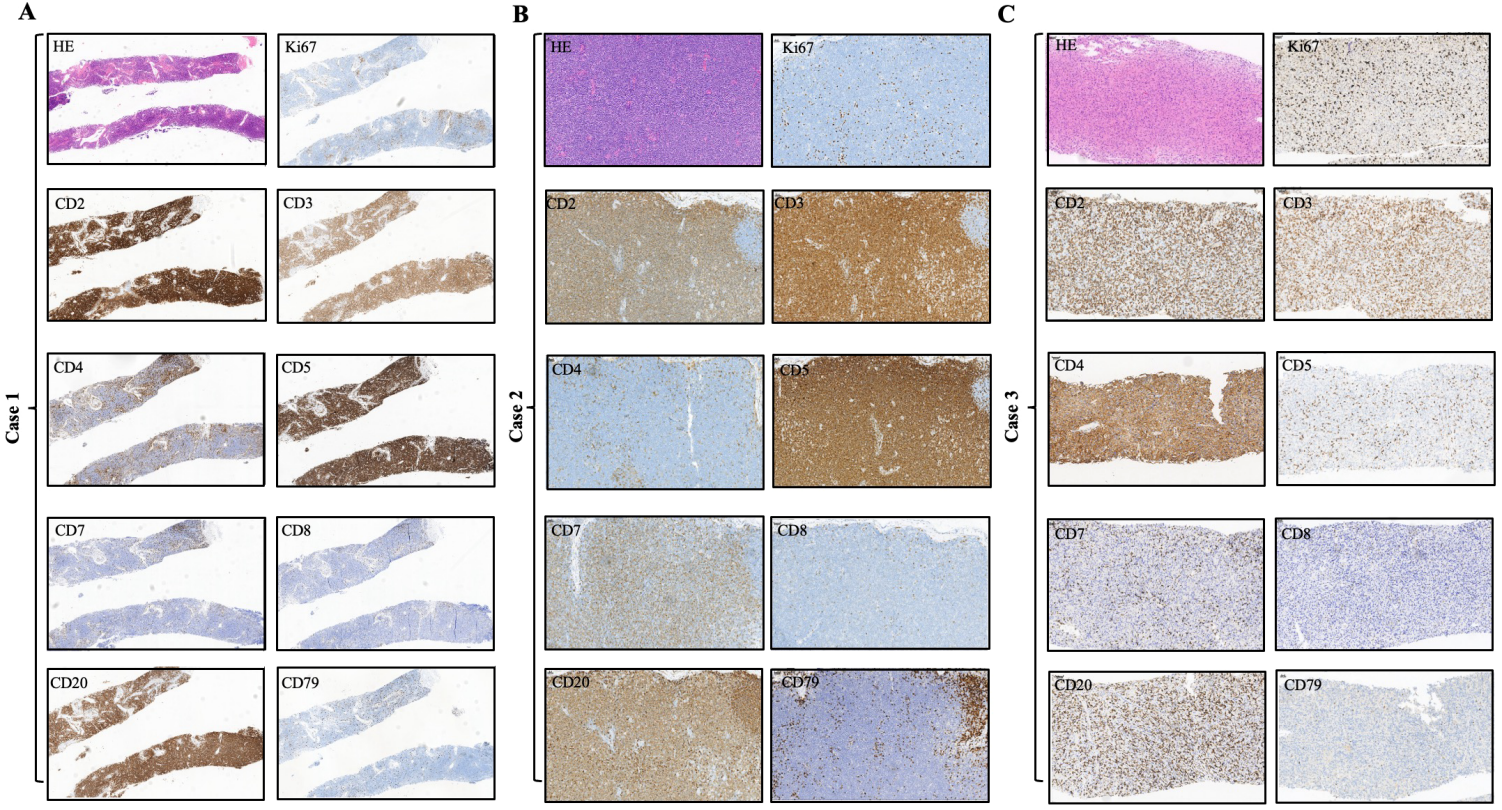
Immunohistochemical findings of the infiltrated lymph nodes in all three CD20+ PTCL-NOS patients. **(A)** Case 1, **(B)** case 2, **(C)** case 3.

We summarized the immunophenotypic features of all three cases in **Table 2** and **Figure 2**. Specifically, the immunophenotypic results of case 1 were as follows: CD2+, CD3+, CD4-, CD5+, CD7-, CD8-, CD56-, CD20+, CD19-, CD22-, CD79-, PAX5-, MUM1-, CD10-, Bcl-6-, CD21-, CD23-, CD30-, Cyclin-D1-, and Ki67 was approximately 10%. The immunophenotypic results of case 2 were as follows: CD2+, CD3+, CD4-, CD5+, CD7+(dim), CD8-, CD56-, CD20+ (mid), CD19-, CD22-, CD79-, PAX5-, MUM1-, CD10-, Bcl-6+(slight), ICOS-, CXCL13-, PD1-, CD21-, CD23-, SOX10-, Cyclin-D1-, CD30+(dim to mid), and Ki67 was approximately 20%. The immunohistochemical stains of case 3 were as follows: CD2+, CD3+, CD4 + (slight), CD5-, CD7-, CD8-, CD56-, CD20+, CD19-, CD22-, CD79-, PAX5-, MUM1+(slight dim), CD10-, Bcl-6+, ICOS-, CXCL13-, PD1-, CD21-, CD23-, SOX10-, Cyclin-D1-, CD30-, and Ki67 was approximately 35%. According to the IHC results, we found that these tumor cells were all positive for the T-cell antigens CD2 and CD3 and negative for CD8 and CD56. Particularly, the lymphoma cells were positive for CD20 while other B-cell antigens such as CD19, CD22, CD79, and PAX5 were all negative. Several neoplastic cells from our cases expressed only one T follicular helper cell-associated marker BCL6, as other markers such as CD10, CXCL13, ICOS, and PD-1 were all negative. Negative CD21 and CD23 staining in all the cases suggest that tumor tissues have unobvious disruption of FDC meshworks, which further excluded the diagnosis of angioimmunoblastic T-cell lymphoma (AITL). The Ki67 index showed that all tumor cells had relatively low proliferative activity. EBV infections were ruled out by negativity for EBER.

Table 2Immunophenotypic features of CD20+ PTCL-NOS as determined by immunohistochemistry.

**Table:** 

	T-cell Antigens							B-cell Antigens					
case	CD2	CD3	CD4	CD5	CD7	CD8	CD56	CD20	CD19	CD22	CD79a	PAX5	MUM1
1	+	+	-	+	-	-	-	+	-	-	-	-	-
2	+	+	-	+	(dim)+	-	-	(mid)+	-	-	-	-	-
3	+	+	(slight) +	-	-	-	-	+	-	-	-	-	(slight dim) +

**Table:** 

	TFH cell Antigens					Other Antigens						
case	CD10	Bcl6	ICOS	CXCL13	PD1	CD21	CD23	CD30	SOX10	Cyclin-D1	Ki67	EBER
1	-	-	-	-	-	-	-	-	-	-	10%	-
2	-	(slight) +	-	-	-	-	-	(dim to mid)+	-	-	20%	-
3	-	+	-	-	-	-	-	-	-	-	35%	-

**Figure 3:**
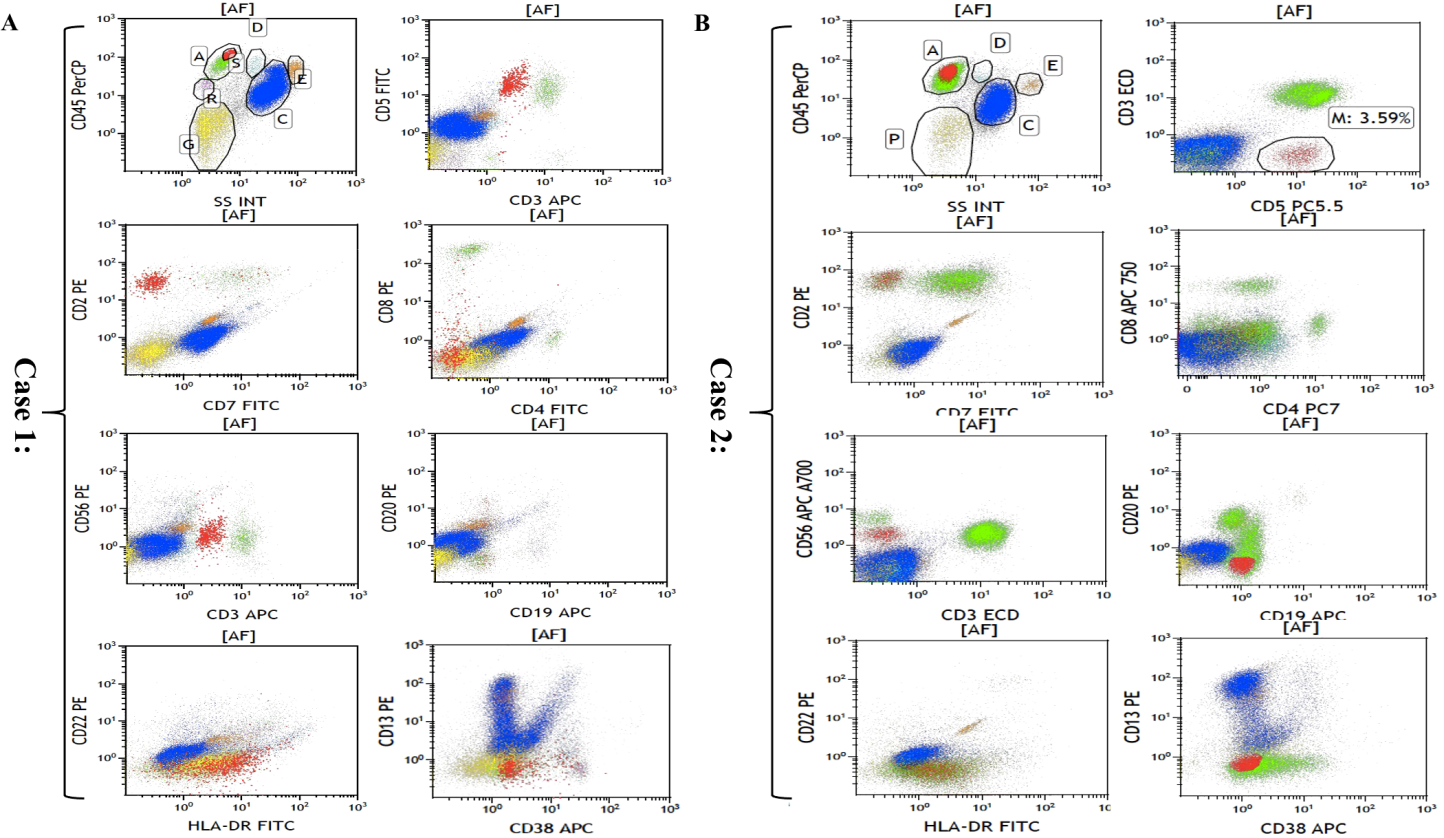
Flow cytometric analyses of bone marrow in case 1 **(A)** and case 2 **(B)**. Cells in red are representative of malignant lymphoma cells.

The flow cytometric analyses of each case are summarized in **Table 3** and **Figure 3**. Although PET-CT displayed an abnormal FDG accumulation in the bone marrow of the left lower tibia, no malignant lymphoma cells were found to have infiltrated by right iliac bone marrow biopsy in case 3. Flow cytometry showed strongly positive T lymphocyte markers (CD2 and CD5), and other T lymphocyte markers, such as CD4, CD7, CD8, and CD56, were negative in the other two cases. As for CD3 expression, case 1 was dim positive while case 2 was negative. Notably, unlike the immunophenotypic features in IHC, neoplastic cells were found to have negatively expressed all B-cell-associated antigens by flow cytometry.

**Table 3:** Immunophenotypic features of CD20+ PTCL-NOS as determined by flow cytometry.

	T-cell antigens							B-cell antigens				Other antigens	
Case	CD2	CD3	CD4	CD5	CD7	CD8	CD56	CD20	CD19	CD22	CD79a	CD13	CD38
1	+	(dim)+	–	+	–	–	–	–	–	–	–	–	–
2	+	–	–	+	–	–	–	–	–	–	–	–	–
3	ND	ND	ND	ND	ND	ND	ND	ND	ND	ND	ND	ND	ND

ND, not detected.

### 2.3 Molecular genetic findings

The results of the molecular genetic studies are summarized in **Table 4**. The assessment of T- and B-cell clonality was performed on the DNA extracted from whole lymph nodes. All the cases showed monoclonal expansion of T-cell receptor (TCR) gene rearrangement, whereas there was no evidence of clonal B-cell population by IgH gene rearrangement. PTCL-NOS is a heterogeneous entity in terms of genetic profile, and the mutational alterations of CD20+ PTCL-NOS are even less clear. Here, we performed genomic profiling of these CD20+ PTCL-NOS patients using a targeted next-generation sequencing panel (Hemasalus™). The somatic mutations that occurred in these cases included DDX3X, MGMT, PIK3CD, CHD8, ARID1A, TET2, FAT4, STAT3, JAK3, PTPRD, PLCG2, PIK3R1, DNMT3A, and KDM2B. Among these mutations, DDX3X and TET2 mutations were present in two cases, and the other gene mutations were present in only one case.

**Table 4:** Molecular genetic findings in CD20+ PTCL-NOS.

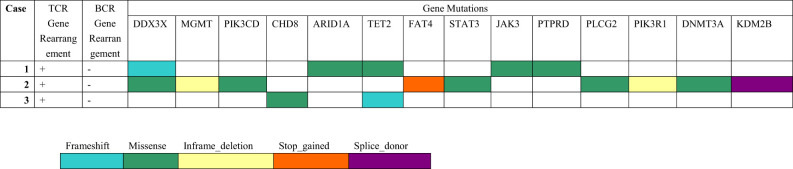

## 3 Discussion

PTCL is a heterogeneous group of lymphoproliferative disorders arising from mature T-cells, mainly including PTCL-NOS (26%), angioimmunoblastic T-cell lymphoma (AITL, 19%), and anaplastic large cell lymphoma (ALCL, 13%) (9). PTCL-NOS represents a vastly heterogeneous disease with great variation in biology, clinical presentation, and outcome (10). The diagnosis of PTCL-NOS is made based on the presence of heterogeneous morphology, an aberrant T-cell immunophenotype (e.g. commonly expressed CD2, CD3, and CD4, and variablely expressed CD4 or CD8) and a clonal TCR gene rearrangement (2, 11). The specific immunomorphology of AITL is characterized by the simultaneous spatiotemporal proliferation of T follicular helper cells (CD4, CD10, Bcl-6, and PD-1), B cells (CD20 and EZH2), and follicular dendritic cells (CD21 and CD23), which can be distinguished from PTCL-NOS (12).

CD20 is a transmembrane protein generally expressed on both benign and neoplastic B lymphocytes and is crucial for B-cell activation, proliferation, and differentiation (13, 14). Small subsets of normal T cells are weakly (dim) CD20 positive, 2/3 are CD8-positive, and 1/3 are CD4-positive. This population of T cells tends to express the γδ TCR gene (15). Likewise, aberrant expression of CD20 has also been described in PTCL (e.g., PTCL-NOS and AITL), albeit at a low incidence (16). This CD20+ PTCL may cause diagnostic errors with B-cell non-Hodgkin’s lymphoma. Generally speaking, CD20+ malignant T lymphoma cells are positive for CD3 and monoclonal TCR gene rearrangements and negative for CD10, CD19, or CD79a and clonal IgH gene rearrangements. The other typical histopathological features of PTCL-NOS are the loss of CD5 and CD7. However, the immunohistology of CD20+ PTCL-NOS we report here showed that 2/3 cases were positive for CD5 and 1/3 cases was positive for CD7. CD20 expression varies from strong to weak in PTCL (17, 18). Several hypotheses have been advanced regarding the origin of CD20 expression in T lymphoma cells. First, these malignant T cells may be transformed from the aforementioned normal CD20+ T cells. Second, the expression of CD20 could be the result of T-cell activation and may represent a truly aberrant phenotype. Third, this may be the result of T lymphocytes trogocytosing CD20 antigen from surrounding B cells (19–21). Several studies have found uniformly dim CD20 expression in CD20+ PTCL cells, supporting the theory that these cells might be derived from a CD20-dim subset of non-neoplastic T-lymphocytes (4). In the cases we described here, the expression of CD20 was mildly to strongly positive, and the immunophenotype was predominantly CD4-CD8-, which may support the self-expression of CD20 by the tumor cells. However, the actual function of CD20 in neoplastic T lymphocytes is still unclear.

Due to PTCL-NOS still being regarded as a heterogeneous category encompassing PTCL cases not fitting any other more homogeneous subtypes, no recurrent driver mutations have been reported thus far (22). Recently, Schatz et al. identified 89 protein-coding mutations in 28 PTCL-NOS cases. The distribution of these gene mutations included epigenetic regulators (MLL2, KDM6A, MLL, TET2, TET1, DMNT3A, and ARID1B), TCR signaling mediators (TNFAIP3, APC, CHD8, and ZAP70), tumor suppressors (TP53 and ATM), and transcription factors (FOXO1 and BCOTL1). They concluded that cases with alterations in histone methylation (MLL2, KDM6A, and MLL) had worse OS than unaffected cases, whereas there was no such effect for either DNA methylation (TET2, DNMT3A, and TET1) or signaling (TNFAIP3, APC, CHD8, ZAP70, NF1, TNFRSF14, and TRAF3) (23). In our study, these CD20+ PTCL-NOS cases harbored mutations including DDX3X, TET2, MGMT, PIK3CD, CHD8, ARID1A, FAT4, STAT3, JAK3, PTPRD, PLCG2, PIK3R1, DNMT3A, and KDM2B. Moreover, DDX3X, MGMT, PIK3CD, ARID1A, FAT4, STAT3, JAK3, PTPRD, PLCG2, PIK3R1, and KDM2B mutations were not detected in the abovementioned non-CD20+ PTCL-NOS patients. Interestingly, Wai et al. discovered that the most mutated gene in a EBV-positive variant of PTCL was TET2, followed by PIK3CD, STAT3, DDX3X, and PTPRD (24). These genomic findings were consistent with the mutated genes demonstrated in our CD20+ PTCL-NOS patients, although all our cases were negative for EBV infection. Whether this genetic similarity was a chance event or a correlation needs to be further investigated. Furthermore, another study found that recurrent gene deletions, including CDKN2A and PTEN, may be relevant for a PTCL-NOS differential diagnosis (22). However, the CD20+ PTCL-NOS patients in our study did not show the deletion of these two genes.

What are the general clinical features of CD20+ PTCL-NOS? According to the limited research, CD20+ PTCL-NOS often presents in elderly patients, more commonly occurs in men, and most cases have nodal lesions, low ECOG-PS scores, rare BM involvement, advanced clinical stage, high International Prognostic Index (IPI) score, and aggressive clinical behavior (3, 4, 18). Furthermore, a multi-center retrospective study found that aberrant CD20 expression was a significantly poor prognostic factor for PFS but not for OS in PTCL (3). In our study, these three patients ranged in age from 48 to 69 years, with a male/female ratio of 2/1, and all had nodal lesions with relatively low ECOG-PS scores. Furthermore, all the cases exhibited Ann Arbor stage IV, had poor PFS but relatively long overall survival. These findings were consistent with the conclusions drawn from previous research. Unexpectedly, the patients we reported here showed frequent BM infiltration but low IPI scores. It is worthwhile to mention that flow cytometry analysis of atypical lymphocytes in tumor-involved lymph nodes showed good consistency with those in bone marrow by previous studies (4). Notably, CD20 was absent in bone marrow infiltrating tumor cells in cases 1 and 2, but whether this phenomenon is generalized or exceptional remains uncertain. RNAseq analysis by Vlaming et al. revealed that CD20-positive T cells had reduced transmigration and an enhanced adhesive profile combined with an enhanced activation status (21). Therefore, we speculate that bone marrow-infiltrating T lymphoma cells are CD20 negative on the basis that neoplastic T cells might mimic similar transmigration characteristics. Further high-throughput sequencing of the sorted tumorigenic T cells from lymph nodes and bone marrow may explain the difference in the future. Our study has several limitations, especially the small sample size and incomplete follow-up data (only 2 years of follow-up of survival), and more multicenter studies are needed to substantiate the general clinical features of CD20+ PTCL-NOS.

Conventional chemotherapy such as CHOP/CHOP-like induction has been the standard therapy for PTCL since the 1970s, but has not adequately prolonged the prognosis (25). The most widely used CHOP+X regimen is CHOPE, which may improve the efficacy and survival in PTCL (26). However, the curative effects of CHOPE chemotherapy as a first-line treatment in our CD20+ PTCL-NOS patients were not satisfactory. After this treatment, one patient subsequently returned to the local hospital, and the other two patients were started on second-line therapy. Case 2 received brentuximab vedotin (BV)-CHP therapy for tumor cells expressing CD30. Case 3 was treated with a GemoxD regimen combined with radiotherapy. Unfortunately, the clinical response of the applied therapies in these two patients remained poor (case 2: SD; case 3: PR). The undesirable responses to the first- and second-line regimens against CD20+ PTCL suggested that additional key drugs are necessary for clinical improvement. New drugs, such as histone deacetylase inhibitors (HDACi), azacytidine, EZH1/2 inhibitors, PI3K inhibitors, and JAK inhibitors, are promising to change the landscape in PTCL-NOS (25). Furthermore, the aberrant expression of CD20 may have therapeutic implications, and anti-CD20 monoclonal antibodies are prospective candidates since the efficacy of rituximab could be associated with the intensity of CD20 expression in T cells and its homogeneity in tumor tissue, and CHOP and CHOP-like regimens are not effective for CD20+ PTCL-NOS (20). Therefore, we tried a therapy with HDACi chidamide combined with azacytidine, alternating with rituximab in combination with a non-CHOP regimen for further treatment in cases 2 and 3. However, these two patients still had poor responses. Thus, we can see that rituximab efficacy remained low even when CD20 intensity was moderately or highly positive in CD20+ PTCL-NOS patients. Mangogna et al. also found that rituximab plus chemotherapy provided no clinical benefit in a CD20/CD79 double positive PTCL-NOS case (17). Whether other anti-CD20 monoclonal antibodies are prospective candidates for CD20+ PTCL-NOS remains unknown. Hence, we concluded that CD20+ PTCL-NOS tends to be less responsive to many traditional chemotherapies and has a poor prognosis even in the era of new drugs. Since all the above are case report findings, further multi-center clinical investigations are necessary to establish a treatment strategy for CD20+ PTCL-NOS.

In summary, our case series of CD20+ PTCL-NOS were characterized by a prevalence in elderly men and late staging with frequent BM metastasis but low IPI scores. Phenotypically, the tumor cells in the lymph nodes were positive for CD2, CD3, and CD20 with CD4/8 double negativity. However, BM-infiltrated malignant T cells did not display CD20 expression. Genomically, CD20+ PTCL-NOS featured a TCR gene rearrangement with frequent DDX3X and TET2 mutations. Although the presence of CD20 was an attractive drug target, the patients tended to be less responsive to rituximab combined chemotherapies. Given the diversity of CD20+ PTCL-NOS, its clinical characteristics, prognostic implications, molecular pathology, and etiology, a proper therapeutic strategy needs to be further elucidated in a large-scale case series.

## Data Availability

The raw data supporting the conclusions of this article will be made available by the authors, without undue reservation.
